# Animal Venoms and Their Components: Molecular Mechanisms of Action V 2.0

**DOI:** 10.3390/toxins16110461

**Published:** 2024-10-28

**Authors:** Yuri Utkin

**Affiliations:** Laboratory of Molecular Toxinology, Shemyakin-Ovchinnikov Institute of Bioorganic Chemistry, Russian Academy of Sciences, 117997 Moscow, Russia; yutkin@yandex.ru or utkin@ibch.ru ; Tel.: +7-495-3366522

The adaptation of living beings to natural environmental conditions occurs as a result of various adaptive mechanisms. Adaptive mechanisms of animals include several forms of adaptation to external environmental conditions: morphological, biochemical, physiological and behavioral (ethological). Examples of morphological adaptation are the fangs and claws of predators, which help them in feeding and defense. As a biochemical adaptation, some animals have acquired the ability to produce venoms, which they also use for defense and attack (hunting). To produce venoms, a special gland has developed in which the biosynthesis of compounds, usually called toxins, occurs. Venoms are complex mixtures of compounds of various natures including proteins, peptides, low-molecular organic compounds, salts, etc. One venom can contain several hundred individual compounds, i.e., toxins. Various toxins act on different organs and systems of living organisms; first of all, these are the nervous, cardiovascular and muscular systems. Thus, toxins that act on the nervous system, or neurotoxins, disrupt different stages of nerve impulse transmission [1,2]. Toxins that affect the cardiovascular system can impair the function of the heart and blood vessels, as well as blood clotting [3,4]. There are highly selective toxins that effectively affect only certain life processes, and there are broad-spectrum toxins that affect several different processes. There is also species selectivity in the action of toxins, i.e., some toxins selectively affect certain animal species.

Although the main task of venoms and toxins is to disrupt normal life processes, in some cases they can have a beneficial effect on the organism. The most studied to date is the use of bee venom to correct various pathological conditions (e.g., [5]). There is also evidence of beneficial effects of scorpion venom and toxins [6]. So, the scorpion venom for quite a long time have been used in traditional Chinese medicine [7].

Although the biological effects of most venoms are quite well studied, in a number of cases, the molecular mechanisms underlying these processes require detailed study. This Special Issue intends to unite papers on the molecular basis of the action of venoms and toxins and contains several articles focused on a modern understanding of the various aspects of the molecular mechanisms underlying the action of animal venoms and their components.

Research on venoms has been carried out for quite a long time, and methods that have already become traditional are applied often. However, along with traditional approaches, more modern methods of separation and analysis are also used, and of these, transcriptomics, proteomics and high-resolution mass spectrometry should be mentioned. Although progress in methods for studying the biological activity of compounds is not so rapid, new procedures and approaches for analyzing the biological effects of venoms and their components are nevertheless proposed. Thus, one of the articles in this Special Issue, namely the paper by Reyes et al. (Contribution 1), proposes an original approach to assess the deleterious effects of venom and one of the toxins from North American pit viper *Crotalus scutulatus scutulatus*. Previously it was shown that cysteine-rich secretory proteins (CRiSP) from North American pit viper venom increase vascular permeability both in vitro and in vivo and induce the acute activation of several adhesion and signaling molecules. In the present work, authors performed a proteomic analysis of peritoneal exudate and extracellular plasma vesicles isolated from BALB/c mice 30 min after the injection of *C. scutulatus scutulatus* venom or its purified CRiSP (Css-CRiSP). It was found that some proteins involved in cell adhesion, cytoskeleton rearrangement, signal transduction, immune responses, and vesicle-mediated transports were significantly upregulated or downregulated. These findings provide greater insight into the pathophysiology of snakebites and allow therapeutic interventions to be more precisely targeted.

As already mentioned, a number of toxins have adverse effects on various processes occurring in animal organisms. These toxins include phospholipases A_2_ (PLA_2_). Two articles in this Special Issue are devoted to the study of these enzymes. The article by Sousa et al. (Contribution 2) discusses the diversity of PLA_2_ in the venom of the common lancehead *Bothrops atrox*. Three distinct PLA_2_s, differing in the levels of induced edema, inflammatory nociception, indirect hemolysis, and anticoagulant activity, were isolated from *B. atrox* venom. The isolated enzymes reacted differently with several antivenoms, with the most toxic PLA_2_ not being recognized or neutralized by the antivenoms used in this study. The authors concluded that the found PLA_2_ diversity may play adaptive roles in the venoms but may also affect human envenomations and have negative effects on their treatment with antivenoms.

To reveal the possible cardiovascular effects of heterodimeric PLA_2_ HDP-1 from the viper *Vipera nikolskii*, in their paper, Averin et al. (Contribution 3) describe the HDP-1 influence on papillary muscle (PM) contractility and the tension of the aortic rings (ARs). It was found that in contrast to monomeric PLA_2_s from snake venoms, which inhibited cardiac function at high concentrations, HDP-1 produced in PMs a stable, long-term, positive inotropic effect, which did not turn into contractures at the concentrations studied. However, in phenylephrine preconstructed ARs, HDP-1 similarly to other PLA_2_s induced a vasorelaxant effect. Although the positive inotropic effect may be beneficial, the possible cardiovascular complications should be kept in mind when treating Nikolsky’s viper bites.

It should be noted that many components of animal venoms affect the functions of the cardiovascular system, but only a small number of them have a beneficial effect. One such component, namely natriuretic-like peptide lebetin 2 (L2) from *Macrovipera lebetina* venom exerting potent heart protection in myocardial infarction, is considered in the article published in this Special Issue by Allaoui et al. (Contribution 4). In this article, the molecular mechanisms underlying its cardioprotective effect were studied using molecular modeling, molecular docking and molecular dynamics. It was found that L2 has a higher affinity for all human natriuretic peptide receptors compared to the B-type natriuretic peptide recombinant form, nesiritide, which had been suggested as a treatment for decompensated heart failure. So, L2 may be considered a good candidate for the development of new, more effective cardioprotective drugs.

As discussed above, venoms and venomous animals have been used in medicine since ancient times. Research currently underway is making it possible to establish the molecular mechanisms underlying the medicinal properties of venoms. One of the papers in this Special Issue, namely the article by Qin et al. (Contribution 5), is devoted to the characterization of the active components of traditional Chinese medical material. In this paper, an important traditional Chinese medical material, which is thermally processed scorpion *Buthus martensii*, was analyzed. This analysis resulted in the identification of a new degraded peptide, BmTX4-P1, which represents a part of the venom-derived wild-type peptide toxin BmTX4. Synthetic and recombinant BmTX4-P1 peptides inhibited human Kv1.2 and Kv1.3 potassium channels. Thus, a method to obtain the biologically active degraded peptides from processed *B. martensii* scorpions was developed.

In conclusion, the papers in this Special Issue demonstrated different approaches to the investigation of animal venoms and their components. Published studies have shown that the molecular mechanisms of action of animal venoms and toxins are of great interest and deserve further extensive and careful investigation. This investigation may result in the development of effective methods for the treatment of bites and stings of venomous animals as well as the creation of new medicines.
